# The Association Between the Gut Microbiota and Parkinson's Disease, a Meta-Analysis

**DOI:** 10.3389/fnagi.2021.636545

**Published:** 2021-02-12

**Authors:** Ting Shen, Yumei Yue, Tingting He, Cong Huang, Boyi Qu, Wen Lv, Hsin-Yi Lai

**Affiliations:** ^1^Department of Neurology of the Second Affiliated Hospital, Interdisciplinary Institute of Neuroscience and Technology, Key Laboratory of Medical Neurobiology of Zhejiang Province, Zhejiang University School of Medicine, Zhejiang University, Hangzhou, China; ^2^Key Laboratory for Biomedical Engineering of Ministry of Education, College of Biomedical Engineering and Instrument Science, Zhejiang University, Hangzhou, China; ^3^Department of Neurology of Sir Run Run Shaw Hospital, Zhejiang University School of Medicine, Zhejiang University, Hangzhou, China; ^4^Department of Sports and Exercise Science, Zhejiang University, Hangzhou, China

**Keywords:** Parkinson's disease, non-motor symptoms, gut-brain axis, gut microbiota, meta-analysis

## Abstract

Patients with Parkinson's disease (PD) were often observed with gastrointestinal symptoms, which preceded the onset of motor symptoms. Neuropathology of PD has also been found in the enteric nervous system (ENS). Many studies have reported significant PD-related alterations of gut microbiota. This meta-analysis was performed to evaluate the differences of gut microbiota between patients with PD and healthy controls (HCs) across different geographical regions. We conducted a systematic online search for case-control studies detecting gut microbiota in patients with PD and HCs. Mean difference (MD) and 95% confidence interval (CI) were calculated to access alterations in the abundance of certain microbiota families in PD. Fifteen case-control studies were included in this meta-analysis study. Our results showed significant lower abundance levels of *Prevotellaceae* (MD = −0.37, 95% CI = −0.62 to −0.11), *Faecalibacterium* (MD = −0.41, 95% CI: −0.57 to −0.24), and *Lachnospiraceae* (MD = −0.34, 95% CI = −0.59 to −0.09) in patients with PD compared to HCs. Significant higher abundance level of *Bifidobacteriaceae* (MD = 0.38, 95%; CI = 0.12 to 0.63), *Ruminococcaceae* (MD = 0.58, 95% CI = 0.07 to 1.10), *Verrucomicrobiaceae* (MD = 0.45, 95% CI = 0.21 to 0.69), and *Christensenellaceae* (MD = 0.20, 95% CI = 0.07 to 0.34) was also found in patients with PD. Thus, shared alterations of certain gut microbiota were detected in patients with PD across different geographical regions. These PD-related gut microbiota dysbiosis might lead to the impairment of short-chain fatty acids (SCFAs) producing process, lipid metabolism, immunoregulatory function, and intestinal permeability, which contribute to the pathogenesis of PD.

## Introduction

Parkinson's disease (PD) is a chronic, progressive, multisystem neurodegenerative movement disorder (Poewe et al., 2017). Patients with PD suffer from characteristic motor symptoms including resting tremor, bradykinesia, rigidity, and gait abnormalities, as well as non-motor symptoms such as hyposmia, sleep disorders, depression, and gastrointestinal (GI) symptoms (Kalia and Lang, 2015). Up to 80% of patients with PD are observed with constipation, the most common GI symptom in PD and are often preceded by the onset of motor symptoms by years (Su et al., 2017). Thus, the constipation symptom is regarded as a clinical biomarker for diagnosing prodromal PD (Berg et al., 2015). The main neuropathological characteristics of PD are loss of dopaminergic neurons in the substantia nigra pars compacta and the presence of Lewy bodies (LBs) or Lewy neurites, which consist of the abnormal α-synuclein aggregates (Abeliovich and Gitler, 2016). Braak staging traced the course of pathology, stating that PD started when a pathogen enters the body *via* the nose or the GI system (Braak et al., 2003), leading to the formation of LBs and spreading from the enteric nervous system (ENS) to the central nervous system (CNS) through the vagus nerve (Rietdijk et al., 2017). Therefore, the role of the “gut-brain axis” started drawing more attention in investigating the pathogenic mechanism of PD.

Higher susceptibility to PD was observed when intestinal infection existed (Huang et al., 2018; Brudek, 2019), which might trigger PD-like symptoms (Matheoud et al., 2019). PD-derived gut microbiota could enhance α-synuclein-mediated motor deficits and brain pathology in a mouse model, while germ-free mouse PD model showed milder α-synuclein pathology (Sampson et al., 2016). Thus, intestinal microbiota disturbance could be considered as a potential risk factor for PD. Gut microbiota is a complex system, producing all sorts of protective compounds and acting as a barrier against pathogens (Nair et al., 2018). Growing evidence has indicated that the abnormality of gut microbiota and its metabolic products may be triggers for the formation of LBs in the ENS. The *Hepatitis C virus* infection was thought to be dopaminergic toxic, which was similar to the effect of 1-methyl-4-phenylpyridinium (MPP+; Wu et al., 2015). The *helicobacter pylori* infection was observed to be associated with an increased risk of PD and worse PD motor severity (Shen et al., 2017). However, although fungal DNA and proteins were detected in post-mortem PD brains, there was no compelling evidence of gut microbiome contribution to PD pathophysiology (Cirstea et al., 2020), which still needs further investigation.

Recently, studies mainly focused on the bacterial component of microbiota in fecal samples. And PD-related alterations of abundance and equilibrium of gut microbiota were reported (Keshavarzian et al., 2015; Hill-Burns et al., 2017; Mertsalmi et al., 2017; Heintz-Buschart et al., 2018; Tetz et al., 2018; Aho et al., 2019; Barichella et al., 2019; Li C. et al., 2019; Li F. et al., 2019; Ren et al., 2020). Significant reduction of several gut microbiota's metabolic products were found in patients with PD, which may contribute to constipation in patients with PD (Unger et al., 2016). Functional differences in β-glucuronate and tryptophan degrading pathways were revealed in patients with PD compared to healthy controls (HCs) (Bedarf et al., 2017). Putative neuroprotective bioactive molecules such as short-chain fatty acids (SCFAs), ubiquinones, and salicylate, as well as neurodegeneration related compounds such as ceramides, sphingosine, and trimethylamine N-oxide, were altered in PD (Tan et al., 2020). Several gut microbiota were also found to be correlated with the clinical characteristics of PD, including disease duration, motor symptom severity, and non-motor symptoms (Qian et al., 2018). In addition, metabolome compositional differences such as the lower SCFAs were associated with poorer cognition, and lower butyrate levels were correlated with worse postural instability gait disorder scores in PD (Tan et al., 2020). A 2-year follow-up study indicated that the total counts of gut microbiota decreased during the course of PD progression and differed between deteriorating and stable PD groups, which may be used as a diagnostic tool for monitoring the progression of PD (Minato et al., 2017). An index was calculated based on 25 gene markers from the gut microbiota that were significantly changed in PD, and this potential diagnostic biomarker had the power to distinguish patients with PD from multiple system atrophy patients (Qian et al., 2020). Furthermore, alterations in the gut microbial activities could possibly lead to heterogeneous responses to levodopa observed among patients with PD, including decreased efficacy and harmful side effects (Maini Rekdal et al., 2019). Therefore, the pathogenesis and clinical manifestations of PD may be related to the dysfunction of the “gut microbiota-gut-brain axis.”

However, the gut microbiota structure varied across different geographical regions, which might lead to inconsistent results. Therefore, in order to verify the shared variations of certain gut microbiota, which presented relatively stable in patients with PD, we performed a meta-analysis to review the alterations of gut microbiota in patients with PD compared to HCs around the world and discussed its possible role in PD.

## Methods

This meta-analysis was performed in accordance with the guidelines of the Meta-analysis Of Observational Studies in Epidemiology (MOOSE) group on meta-analyses of observational studies (Stroup et al., 2000), and also referenced the Preferred Reporting Items for Systematic Reviews and Meta-analyses (PRISMA) statement (Liberati et al., 2009).

### Literature Search

In order to identify relevant studies of gut microbiota analysis in PD for this meta-analysis, a systematic literature search was conducted using the following English and Chinese databases (up to August 2020): PubMed, Web of Science, Chinese National Knowledge Infrastructure (CNKI) databases, and Wanfang database. The search strategy to identify all potential studies involved using combinations of the following terms: (Parkinson's disease OR Parkinson disease OR Parkinsonism) AND (microbes OR microbiome OR microbiota OR bacteria) in Title/Abstract. We also manually searched the references cited in the selected articles or reviews to identify additional relevant studies.

### Study Selection

The inclusion criteria for this meta-analysis were as follows: (1) gut microbiota studies comparing patients with PD with HCs; (2) fecal samples; (3) microbiota abundance being expressed as mean proportions of each microbiota; and (4) ability to obtain the mean difference (MD) with 95% confidence interval (CI) in these two groups or sufficient data to calculate these. Studies were excluded if they were any of the following: (1) failure to finally obtain sufficient data; (2) duplicate data reported in other studies; and (3) case-only studies, family-based studies, intervention studies, and review articles. Two investigators (Shen and Yue) jointly screened for eligible studies by reading the titles and abstracts of identified studies, then carefully reviewed full articles of the rest studies, and excluded studies not meeting the inclusion criteria.

### Data Extraction

Two investigators (Shen and Yue) independently extracted data from included studies including the following items: (1) general information (author, year of publication, and location); (2) patient characteristics (gender and age); (3) experimental methods (diagnostic criteria, sample size, and microbiology assessment technique); and (4) effect size of microbiota abundance (MD and 95% CI). Graphs and plots were also common forms of data reports. Because some articles showed relevant data indirectly, the software GetData Graph Digitizer 2.25 (http://getdata-graph-digitizer.com/) was applied to digitize and extract sufficient data (Fedorov, 2012; Liao et al., 2015; Tang et al., 2015).

### Quality Assessment

Three investigators (Shen, Yue, and He) independently rated the quality of the included studies. The nine-star Newcastle-Ottawa Quality Assessment Scale (NOS) for case-control studies was used to assess the methodological quality of the included studies (Stang, 2010). Disagreements were solved through discussion or involvement of a third investigator, if necessary. The NOS scale includes three criteria: selection, comparability, and exposure. The selection criteria included four items: (1) adequate case definitions, (2) the representativeness of the cases, (3) the selection of controls, and (4) the definition of controls. The comparability criteria included one item: control for an important factor. The exposure criteria included three items: (1) the ascertainment of exposure, (2) the same method of ascertainment for cases and controls, and (3) the non-response rate.

### Statistical Analysis

Statistical analysis was performed by Review Manager 5.3 software to compare the abundance level of some gut microbiota in patients with PD with HCs if the number of studies for a single bacterium was five or more. Included studies provided means for continuous variables, and we calculated mean difference (MD) and 95% confidence interval (CI) of microbiota abundance as summary statistics. The heterogeneity between studies was assessed using the I^2^ statistics. An I^2^ value closer to 0% was considered as low heterogeneity, I^2^ > 50% was considered to indicate substantial heterogeneity, and I^2^ value closer to 100% was considered as large heterogeneity. Data analysis was performed using the fixed-effect model with low significant heterogeneity or using a random-effect model with substantial and large heterogeneity (I^2^ > 50%). The level of significance was set at *p* < 0.05. A funnel plot was applied to estimate the potential publication bias. Asymmetry of funnel plots indicates significant heterogeneity between selected studies, which lead to publication bias.

## Results

### Study Selection and Characteristics

**Figure 1:**
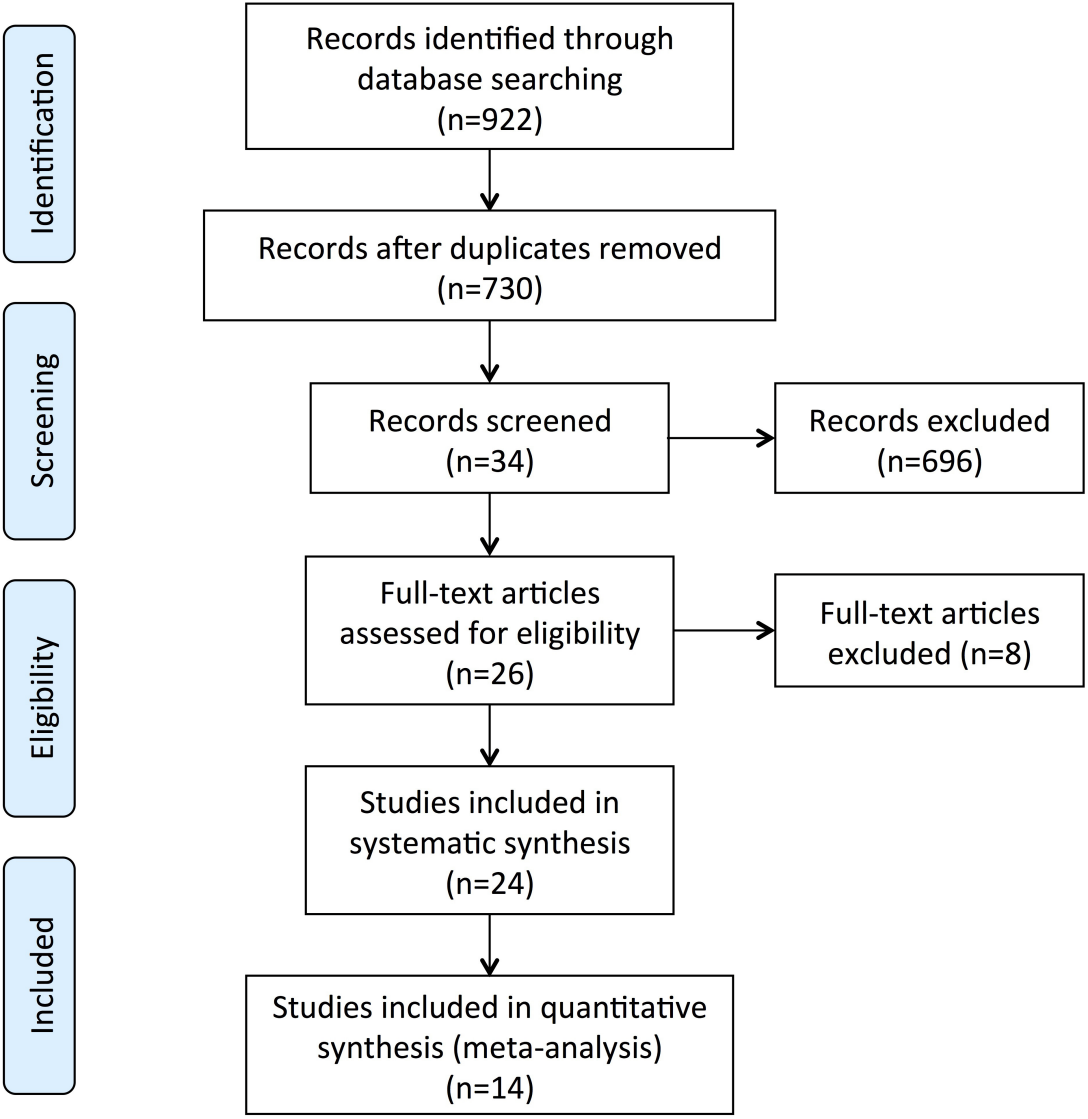
Flow diagram of the assessment of studies identified in this meta-analysis.

The screening process is summarized in a flow diagram (Figure 1). The literature database searches yielded 922 potentially relevant records, of which, 192 duplicates were removed, and 696 obviously irrelevant publications were excluded after the review of titles and abstracts. After a further review of full texts, eight records were excluded due to lack of control group, incomplete data, or non-fecal samples. Of the 24 records that were included in the systematic review, 14 studies were included in this meta-analysis after removing nine studies that were not able to provide quantitative data about gut microbiota abundance at the family level and one study that had unmatched age between two groups. The main characteristics of these studies are summarized in Table 1. All the 14 studies were observational case-control studies and were considered relatively high quality according to the NOS scale (Table 2). These studies were conducted in the USA (Hill-Burns et al., 2017), Finland (Scheperjans et al., 2015; Aho et al., 2019), Germany (Unger et al., 2016; Bedarf et al., 2017; Hopfner et al., 2017; Heintz-Buschart et al., 2018), Russia (Petrov et al., 2017), China (Li et al., 2017; Lin et al., 2018; Li C. et al., 2019; Li F. et al., 2019; Ren et al., 2020), and Italy (Barichella et al., 2019). This meta-analysis involved 959 patients with PD and 744 HCs. The mean age of included patients ranged from 62.2 to 76.5 years old, and the number of female subjects ranged from 0.00 to 76.9%. All the 14 included studies obtained gut microbiota from a fecal sample, one of them evaluated microbiota abundance through quantitative polymerase chain reaction (qPCR), and the other 13 studies used the next-generation sequencing (NGS) technique.

**Table 1:** Characteristics of 14 studies included in the systematic review.

**References**	**Location**	**PD case/HCs**			**Experimental Methods**	
		**Number**	**Mean Age ± SD**	**Female Ratio (%)**	**Sample**	**Technique**
Scheperjans et al. (2015)	Finland	72/72	65.3 ± 5.5/64.5 ± 6.9	48.6/50.0	Feces	NGS
Unger et al. (2016)	Germany	34/34	67.7 ± 8.9/64.6 ± 6.6	29.4/47.1	Feces	qPCR
Hill-Burns et al. (2017)	USA	197/130	68.4 ± 9.2/70.3 ± 8.6	33.0/60.8	Feces	NGS
Hopfner et al. (2017)	Germany	29/29	69.2 ±6.5/69.4 ± 6.7	20.7/55.2	Feces	NGS
Bedarf et al. (2017)	Germany	31/28	64.8 ± 9.5/65.6 ± 10.4	0/0	Feces	NGS
Petrov et al. (2017)	Russia	89/66	67.4 ± 2.4/64.5 ± 3.0	-	Feces	NGS
Li et al. (2017)	China	24/14	73.8 ± 6.3/74.6 ± 5.6	33.3/57.1	Feces	NGS
Lin et al. (2018)	China	75/45	60.5 ± 10.7/63.2 ± 6.0	34.7/48.9	Feces	NGS
Heintz-Buschart et al. (2018)	Germany	76/78	68.0 ± 9.7/68.4 ± 6.7	34.0/41.0	Feces	NGS
Barichella et al. (2019)	Italy	193/113	67.6 ± 9.7/65.9 ± 9.9	40.4/58.4	Feces	NGS
Li C. et al. (2019)	China	51/48	62.4 ± 8.2/62.2 ± 9.2	37.3/60.4	Feces	NGS
Li F. et al. (2019)	China	10/10	76.5 ± 7.1/79.5 ± 7.6	30.0/50.0	Feces	NGS
Aho et al. (2019)	Finland	64/64	65.2 ± 5.5/64.5 ± 6.9	48.4/50.0	Feces	NGS
Ren et al. (2020)	China	14/13	60.0 ± 9.2/63.0 ± 8.8	76.9/28.6	Feces	NGS

*PD, Parkinson's disease; HCs, healthy controls; SD, standard deviation; NGS, next-generation sequencing; qPCR, quantitative polymerase chain reaction. “–” means not mentioned in that study*.

**Table 2:** Scores of the 14 studies included in this meta-analysis based on NOS.

**References**	**Selection**				**Comparability**	**Exposure**			**Total Score**
	**Adequate Definition of Cases**	**Representativeness of Cases**	**Selection of Controls**	**Definition of Controls**	Control for Important Factor^*^	**Ascertainment of Exposure**	**Same Method to Ascertain for Cases and Controls**	**Non-response Rate**	
Scheperjans et al. (2015)	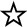	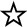	–	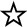	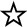	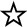	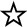	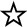	7
Unger et al. (2016)	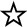	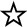	–	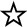	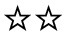	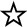	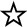	–	7
Hill-Burns et al. (2017)	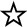	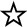	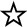	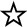	–	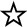	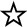	–	6
Hopfner et al. (2017)	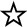	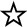	–	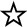	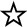	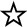	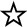	–	6
Bedarf et al. (2017)	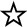	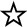	–	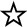	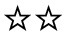	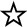	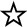	–	7
Petrov et al. (2017)	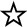	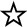	–	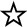	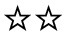	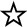	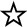	–	7
Li et al. (2017)	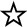	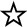	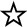	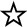	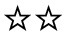	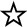	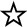	–	8
Lin et al. (2018)	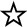	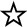	–	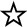	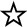	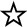	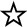	–	6
Heintz-Buschart et al. (2018)	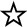	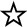	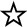	–	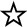	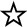	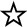	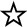	7
Barichella et al. (2019)	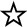	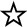	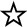	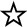	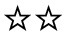	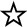	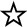	–	8
Li C. et al. (2019)	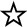	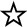	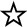	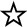	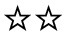	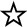	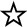	–	8
Li F. et al. (2019)	–	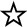	–	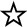	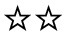	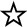	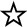	–	6
Aho et al. (2019)	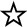	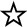	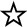	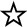	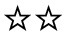	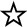	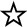	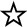	9
Ren et al. (2020)	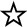	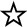	–	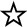	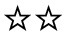	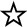	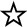	–	7

* *A maximum of two stars can be allotted in this category, one for Age and Sex, the other for other controlled factors*.

### Meta-Analysis of Standardized Mean Difference

We extracted continuous data from included studies to conduct the meta-analysis. We analyzed the alterations of the abundance of *Prevotellaceae, Bifidobacteriaceae, Lactobacillaceae, Faecalibacterium, Ruminococcaceae, Verrucomicrobiaceae, Enterobacteriaceae, Bacteroidaceae, Christensenellaceae*, and *Lachnospiraceae* in patients with PD compared to HCs in the present study. A fixed-effect model was used to evaluate the alterations of *Faecalibacterium, Bacteroidaceae*, and *Christensenellaceae* families due to low heterogeneity between studies, while a random-effect meta-analysis was performed in evaluating the abundance of *Prevotellaceae, Bifidobacteriaceae, Lactobacillaceae, Ruminococcaceae, Verrucomicrobiaceae, Enterobacteriaceae*, and *Lachnospiraceae* families due to substantial and large heterogeneity.

**Figure 2:**
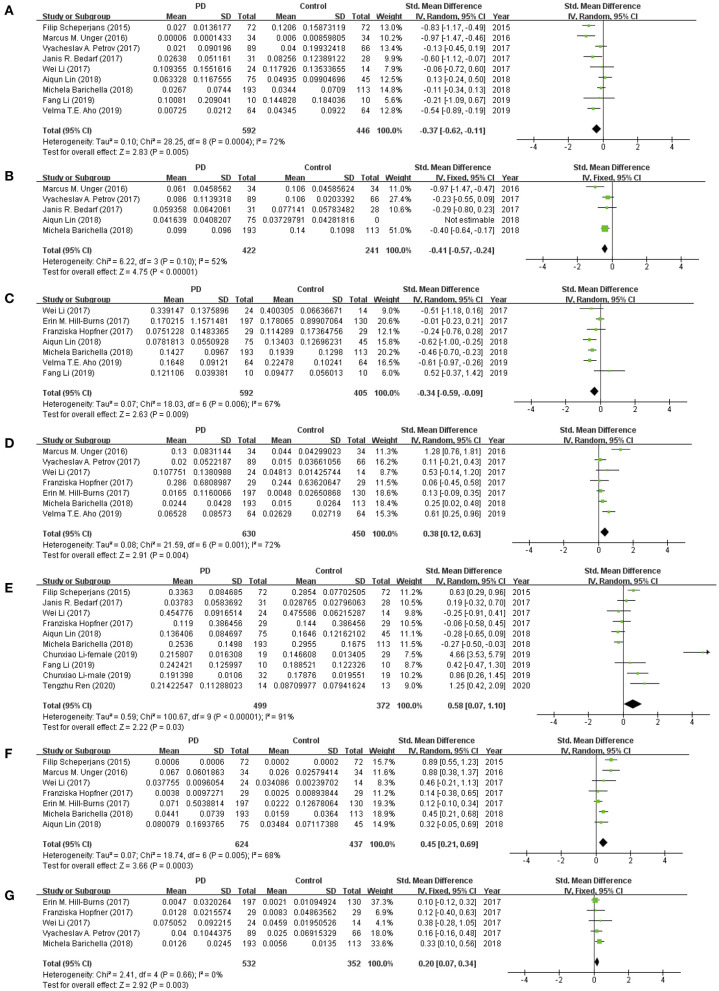
Forest plots of alterations of gut microbiota in patients with Parkinson's disease (PD) vs. healthy controls (HCs): **(A)** *Prevotellaceae*, **(B)** *Faecalibacterium*, **(C)** *Lachnospiraceae*, **(D)** *Bifidobacteriaceae*, **(E)** *Ruminococcaceae*, **(F)** *Verrucomicrobiaceae*, **(G)** *Christensenellaceae*.

This meta-analysis showed significant lower abundance levels of *Prevotellaceae* (MD = −0.37, 95% CI = −0.62 to −0.11; I^2^ = 72%; *p* = 0.005; nine studies; Figure 2A), *Faecalibacterium* (MD = −0.41, 95% CI = −0.57 to −0.24; I^2^ = 52%; *p* < 0.00001; five studies; Figure 2B), and *Lachnospiraceae* (MD = −0.34, 95% CI = −0.59 to −0.09; I^2^ = 67%; *p* = 0.009; seven studies; Figure 2C) in patients with PD compared to HCs. Significant higher abundance level of *Bifidobacteriaceae* (MD = 0.38, 95% CI = 0.12 to 0.63; I^2^ = 72%; *p* < 0.004; seven studies; Figure 2D), and *Ruminococcaceae* (MD = 0.58, 95% CI = 0.07 to 1.10; I^2^ = 91%; *p* < 0.03; nine studies; Figure 2E), *Verrucomicrobiaceae* (MD = 0.45, 95% CI = 0.21 to 0.69; I^2^ = 68%; *p* = 0.0003; seven studies; Figure 2F), and *Christensenellaceae* (MD = 0.20, 95% CI = 0.07 to 0.34; I^2^ = 0%; *p* = 0.003; seven studies; Figure 2G) was found in patients with PD. The differences of abundance of *Lactobacillaceae, Enterobacteriaceae*, and *Bacteroidaceae* between the two groups were not statistically significant (*p* > 0.05).

### Analysis of Publication Bias

**Figure 3:**
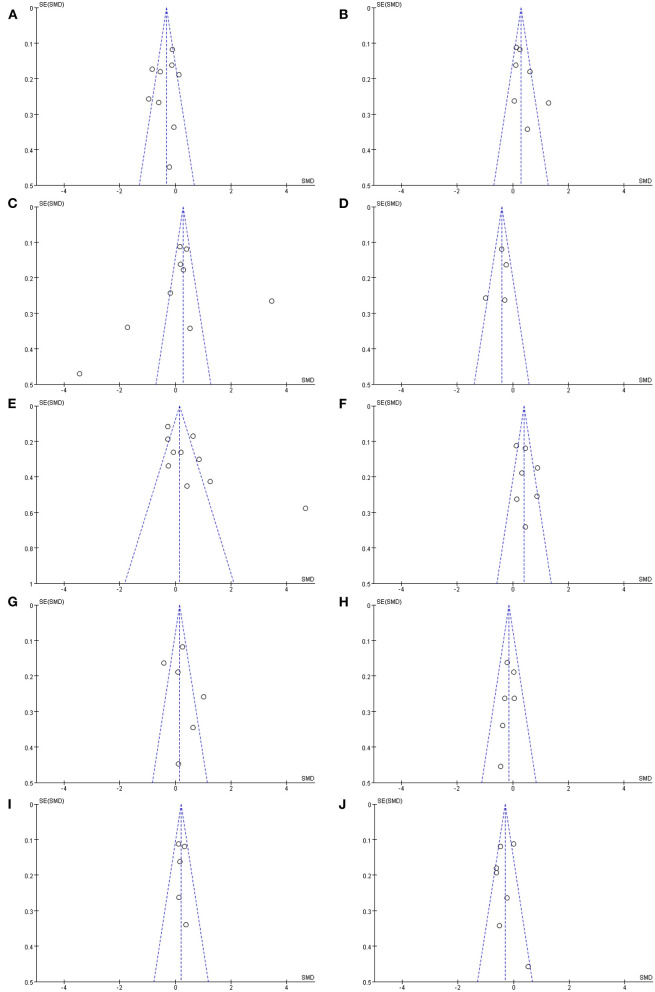
Funnel plots of included studies analyzing alterations of gut microbiota in patients with PD vs. HCs: **(A)** *Prevotellaceae*, **(B)** *Bifidobacteriaceae*, **(C)** *Lactobacillaceae*, **(D)** *Faecalibacterium*, **(E)** *Ruminococcaceae*, **(F)** *Verrucomicrobiaceae*, **(G)** *Enterobacteriaceae*, **(H)** *Bacteroidaceae*, **(I)** *Christensenellaceae*, **(J)** *Lachnospiraceae*.

The publication biases were detected with funnel plots (Figure 3). The funnel plots of *Prevotellaceae* (Figure 3A), *Bifidobacteriaceae* (Figure 3B), *Lactobacillaceae* (Figure 3C), *Ruminococcaceae* (Figure 3E), *Verrucomicrobiaceae* (Figure 3F), *Enterobacteriaceae* (Figure 3G), and *Lachnospiraceae* (Figure 3J) suggested possible bias, which indicated significant heterogeneity between the selected studies. Besides, the shape of funnel plots of *Faecalibacterium* (Figure 3D), *Bacteroidaceae* (Figure 3H), and *Christensenellaceae* (Figure 3I) showed no obvious asymmetry, which indicated that there was no significant heterogeneity between these studies, and the pooled results were not influenced by publication bias. However, the result of publication bias analysis might be not sufficiently reliable due to the limited number of included studies.

## Discussion

Recent studies have reported gut microbiota alterations and intestinal metabolism abnormality in patients with PD, which may affect brain activity through the microbiota-gut-brain axis (Mayer et al., 2015). However, there is a certain degree of difference between the different research results. Since it was not easy to obtain available original datasets of gene sequencing, one previous meta-analysis was conducted based on only five studies. The abundance of four genera including *Akkermansia, Roseburia, Faecalibacterium*, and *Lachnospiraceae ND3007 group*, as well as one family *Akkermansiaceae*, was found to be changed in PD (Nishiwaki et al., 2020). We attempted to include as many studies as possible, thus we extracted analyzed results of microbiota abundance instead of obtaining the original datasets (Prosberg et al., 2016; Zhuang et al., 2017). This meta-analysis was performed to evaluate the differences of gut microbiota between patients with PD and HCs based on 14 case-control studies. We observed that significantly lower abundance levels of *Prevotellaceae, Faecalibacterium*, and *Lachnospiraceae* in patients with PD compared to HCs, therefore they might be potentially “beneficial” microbiota against PD. Moreover, the families of *Bifidobacteriaceae, Ruminococcaceae, Verrucomicrobiaceae*, and *Christensenellaceae* showed increased abundances in PD. Our results covered discoveries of the previous meta-analysis, and further reported a wider range of altered gut microbiota in patients with PD.

The family *Prevotellaceae* helps the breakdown of carbohydrates from dietary fiber and produces SCFAs, which modulates the activity of ENS and helps to maintain gut homeostasis (Unger et al., 2016; Bedarf et al., 2017; Nair et al., 2018). Decreased abundance of *Prevotellaceae* has been revealed to be associated with decreased levels of the gut hormone ghrelin (Queipo-Ortuno et al., 2013; Scheperjans et al., 2015), which participates in regulating dopaminergic neuron function in the substantia nigra pars compacta and may fight against neurodegeneration in PD (Bayliss et al., 2011; Scheperjans et al., 2015). It has been reported that *Prevotellaceae* was associated with the Unified Parkinson Disease Rating Scale (UPDRS)-III score evaluating the severity of PD (Scheperjans et al., 2015), and the abundance of *Prevotellaceae* obviously decreased along with the progress of PD (Minato et al., 2017). Therefore, the reduction of *Prevotellaceae* could be regarded as a biomarker for PD.

The family *Faecalibacterium* also displays an important role in producing SCFAs and anti-inflammatory metabolites that helps to maintain gut health (Ferreira-Halder et al., 2017). Reduction of *Faecalibacterium* might impair the gut-barrier function and make the ENS more susceptible to infection of enteric pathogens and increase the risk of α-synuclein formation in the ENS (Unger et al., 2016). The abundance of *Faecalibacterium* was negatively correlated with UPDRS score and PD duration, which indicated that it might be related to the development and neuropathology of PD (Li et al., 2017).

Similarly, the family *Lachnospiraceae* also participates in producing beneficial butyrate, which could help to maintain the gut epithelium (Hill-Burns et al., 2017; Lin et al., 2018). The lower abundance of *Lachnospiraceae* might lead to the aggravation of gut inflammation, increased production of toxic substances, and impairment of the gut epithelial barrier (Lin et al., 2018; Barichella et al., 2019). Especially in patients with PD, the reduced abundance of *Lachnospiraceae* was reported to correlate with longer PD disease duration (Keshavarzian et al., 2015; Barichella et al., 2019), cognitive decline, and worse motor symptoms (Barichella et al., 2019). And the use of catechol-O-methyltransferase (COMT) inhibitors might influence the level of *Lachnospiraceae* (Barichella et al., 2019). It is thus clear that the lack of these “beneficial” microbiota might contribute to the pathophysiology of PD. At the same time, anti-Parkinson medication might affect the gut epithelium.

In regard to the family *Bifidobacteriaceae*, a kind of important dominant probiotic participates in several physiological functions including the inhibition of the overgrowth of harmful gut bacteria, the improvement of gut ecological environment, and immune regulation (Hsieh et al., 2020). In PD mouse models, long-term administration of probiotics had neuroprotective effects on dopamine neurons in the substantia nigra and further attenuated motor impairments in gait pattern, balance function, and motor coordination (Hsieh et al., 2020). In patients with PD, probiotics administration also could reduce UPDRS score and improve motor function (Tamtaji et al., 2019). The *Bifidobacteriaceae* family should be “beneficial” microbiota. However, all seven studies reported consistent results that the abundance of *Bifidobacteriaceae* was increased in patients with PD, which might indicate potential compensatory regulation to reconstruct gut homeostasis (Wallen et al., 2020).

The family *Ruminococcaceae* is considered an important cellulose-degrading bacteria that also produce SCFAs. Findings for this gut microbiota in PD were mixed, which might be due to the disease duration. The increased abundance of *Ruminococcaceae* was found to correlate with longer PD disease duration (Hill-Burns et al., 2017). However, the abundance was only increased in patients who had PD for over 10 years, but not in the first 10 years of disease (Hill-Burns et al., 2017). And the use of COMT inhibitors might reduce the level of *Ruminococcaceae* (Barichella et al., 2019), which would also influence the structure of gut microbiota.

And the family *Verrucomicrobiaceae*, one of its main genera, the mucus-degrading bacterium *Akkermansia*, converts mucin to SCFAs that may mediate the immunoregulatory effects. Moreover, it is also involved in proinflammatory pathways, due to its mucus-degrading feature, which leads to the breakdown of the gut-barrier and increased exposure of resident immune cells to pathogens (Jangi et al., 2016; Fujio-Vejar et al., 2017), and thereby causes abnormal aggregation of α-synuclein formation in ENS. Furthermore, the increased level of *Akkermansia* might accelerate the progression of PD (Nishiwaki et al., 2020). In our meta-analysis, a significantly higher abundance level of *Verrucomicrobiaceae* was found in patients with PD, which might link to the development of PD. Similarly, a significant trend effect for disease duration on the increasing abundance of *Verrucomicrobiaceae* was also found in PD (Barichella et al., 2019).

The family *Christensenellaceae* may also play an important role in human health, and its abundance was inversely related to host body mass index (BMI) and visceral fat mass (Waters and Ley, 2019). Since previous studies found that patients with PD were three times more likely than HCs to have a low BMI (Suzuki et al., 2020), and progressive weight loss was commonly seen in PD, with greater loss of both visceral and subcutaneous fat (Yong et al., 2020). Accordingly, all five studies consistently reported a higher abundance of *Christensenellaceae* in patients with PD, indicating that *Christensenellaceae* might play a role in lipid metabolism and provided evidence that an increase of certain gut microbiota would lead to weight loss through influencing lipid absorption. As previously reported, some of the non-motor manifestations in PD were caused by intestinal dysbiosis (Hasuike et al., 2020). Thus, the increased abundance of the family *Christensenellaceae* was also found to be correlated with worse non-motor symptoms (Barichella et al., 2019).

**Figure 4:**
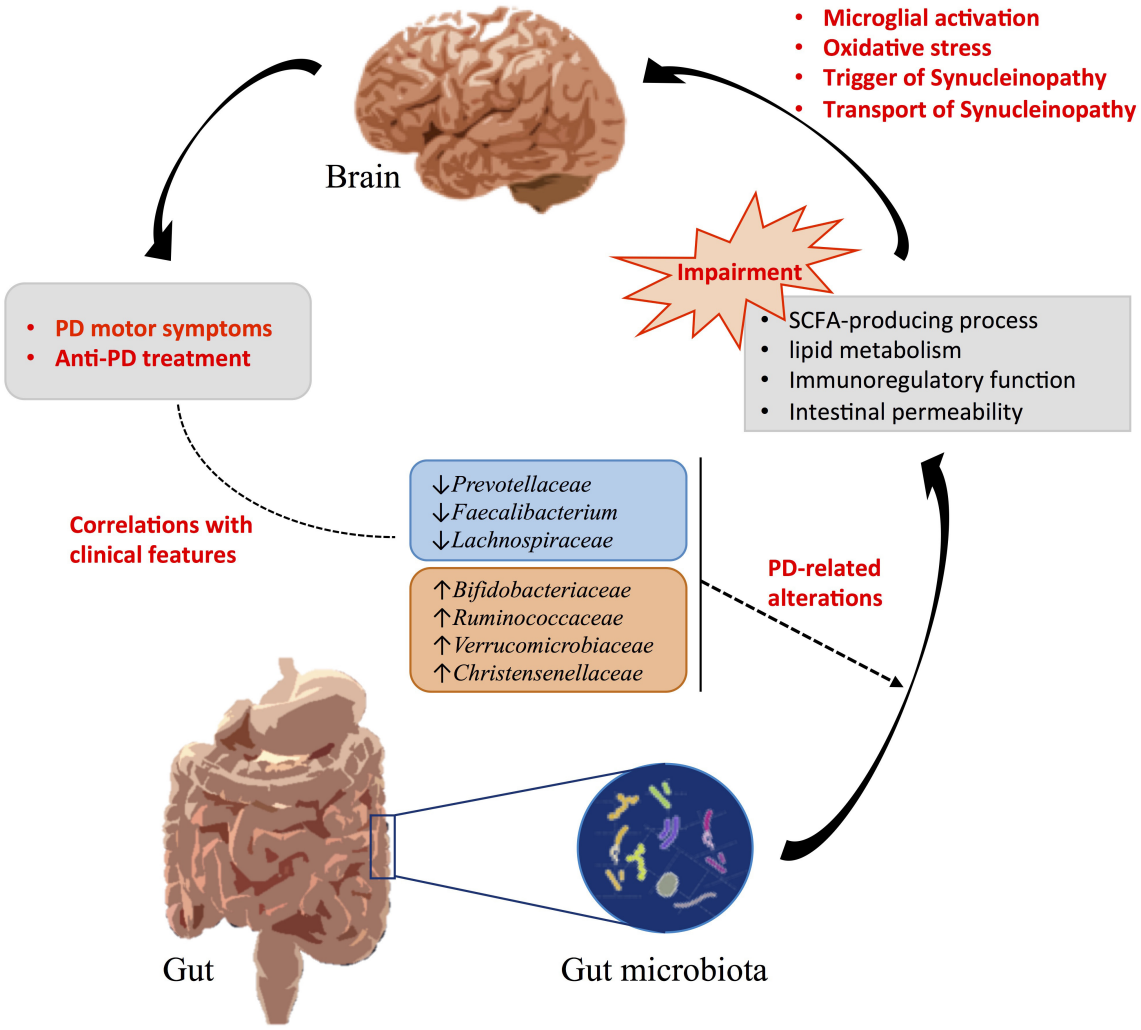
Influences of gut microbiota alterations in PD. PD-related gut microbiota dysbiosis might be related to the clinical features and contribute to triggering synucleinopathy by impairing the “gut microbiota-gut-brain axis” in PD.

Gut microbiota structure varied within individuals due to a series of factors including the mode of delivery, infant feeding, dietary habits, culture, geographical region, age, gender, and so on (Zhuang et al., 2017). But even after taking into account these factors, we found shared changes of abundance levels of *Prevotellaceae, Faecalibacterium, Lachnospiraceae, Bifidobacteriaceae, Verrucomicrobiaceae*, and *Christensenellaceae* in patients with PD across different studies conducted in different geographical regions. Thus, these alterations of gut microbiota were mainly attributed to PD disease statues or might be promotion factors for the progression of PD. Inflammation has been proved to be linked to the development of PD, which activates microglia that play a cruel role in the damage of dopaminergic neurons and aggregation of α-synuclein (Baizabal-Carvallo and Alonso-Juarez, 2020). SCFAs have potential anti-inflammatory and anti-oxidant properties, which might help to regulate neuroinflammation and gut permeability and rescue neuronal damage (Bullich et al., 2019). Imbalance of SCFAs-producing bacteria may cause microglial activation and increased risk of α-synuclein deposition in PD. Regarding the lipid metabolism pathway, lipid dysregulation might be involved in promoting PD pathophysiological processes through oxidative stress and inflammation reaction (Hu et al., 2020). Lipids interact with α-synuclein and further affect the aggregation of α-synuclein and transport of synucleinopathy (Hu et al., 2020; Mori et al., 2020). Thus, altered gut microbiota that participates in lipid metabolism may also contribute to PD pathology. Based on this accumulated information, we suggested that these reported PD-related gut microbiota dysbiosis might contribute to triggering synucleinopathy by impairing the “gut microbiota-gut-brain axis” in PD (Figure 4). Future researches could be further conducted to clarify the cause and effect between gut microbiota and brain pathology, the detailed roles in PD disease progression, and the potential therapeutic targets (Wallen et al., 2020).

However, there are still some limitations in our meta-analysis. Firstly, statistical heterogeneities existed among the included studies, which could be explained by the differences in sample size, geographical regions, study methodology, and criteria of PD. Secondly, it is difficult to obtain raw data from all the included studies, and we used the software GetData Graph Digitizer to digitize and extract sufficient data from graphs and plots of several studies, which might cause another outcome bias. In addition, we only discussed the structure and composition of gut microbiota, and not the transcriptomics and proteomics studies that would provide a deeper understanding of gut microbiota function. These all need to be improved in future studies.

## Conclusion

We reported shared alterations of certain gut microbiota in patients with PD compared to HCs across different geographical regions. Significant lower abundance levels of *Prevotellaceae, Faecalibacterium*, and *Lachnospiraceae*, and higher abundance levels of *Bifidobacteriaceae, Ruminococcaceae, Verrucomicrobiaceae*, and *Christensenellaceae* in patients with PD were observed. The ecological imbalance of these gut microbiota might lead to the impairment of the SCFA-producing process, lipid metabolism, immunoregulatory function, intestinal permeability, etc. Thus, the alteration of the gut microbiota could be considered as an environmental trigger of the PD pathological process and contribute to the development of PD. In future work, a large sample study, as well as metagenomics and metabonomics techniques, are needed to further evaluate the effect of gut microbiota on the development of PD.

## Data Availability Statement

The original contributions presented in the study are included in the article/supplementary material, further inquiries can be directed to the corresponding author/s.

## Author Contributions

TS, YY, and H-YL conceived and designed the study. TS, YY, and TH contributed to data collection and analysis. TS and YY wrote the original draft of the manuscript. WL and BQ provided the technical support. CH and H-YL revised and finalized the manuscript. All authors contributed to the article and approved the submitted version.

## Conflict of Interest

The authors declare that the research was conducted in the absence of any commercial or financial relationships that could be construed as a potential conflict of interest.
